# Modified response evaluation criteria in solid tumors is superior to response evaluation criteria in solid tumors for assessment of responses to sorafenib in patients with advanced hepatocellular carcinoma

**DOI:** 10.1186/s13104-015-1565-2

**Published:** 2015-10-26

**Authors:** Juichi Takada, Hisashi Hidaka, Takahide Nakazawa, Masaaki Kondo, Kazushi Numata, Katsuaki Tanaka, Kotaro Matsunaga, Chiaki Okuse, Satoshi Kobayashi, Manabu Morimoto, Shinichi Ohkawa, Wasaburo Koizumi

**Affiliations:** Department of Gastroenterology, Internal Medicine, Kitasato University Hospital, Kitasato University School of Medicine, 1-15-1, Kitasato, Minami-ku, Sagamihara, Kanagawa 252-0375 Japan; Department of Gastroenterological Center, Yokohama City University Medical Center, Yokohama, Japan; Department of Gastroenterology and Hepatology, St Marianna University School of Medicine, Kawasaki, Japan; Hepatobiliary and Pancreatic Medical Oncology, Kanagawa Cancer Center Hospital, Yokohama, Japan

**Keywords:** Hepatocellular carcinoma, Sorafenib, RECIST, Modified RECIST

## Abstract

**Background:**

Modified response evaluation criteria in solid tumors (mRECIST) and RECIST are used to assess the effect of treatment with targeted agents for hepatocellular carcinoma (HCC). The aim of this study was to determine which set of criteria is superior in patients with advanced HCC treated with sorafenib.

**Methods:**

A multicenter retrospective study to assess the tumor response and patient prognosis of 191 patients with HCC who had been treated with sorafenib from May 2009 through December 2011. We analyzed tumor responses as shown by contrast-enhanced computed tomography scan images according to RECIST 1.1 and mRECIST and compared the findings.

**Results:**

The median duration of follow-up was 9.7 months and median overall survival was 10.8 months. Twenty-five patients (13.1 %) were assessed as responders by mRECIST and 15 (7.8 %) by RECIST 1.1. There was a significant difference in overall survival (OS) between responders and non-responders according to mRECIST (*P* = 0.0117), but no significant difference in OS between responders and non-responders according to RECIST 1.1 (*P* = 0.0722). Sixteen patients (8.4 %) had no measurable enhanced target lesions that could be assessed as required by mRECIST; however, these patients could be assessed by RECIST 1.1. According to RECIST 1.1, eight of them had stable disease (SD) and eight had progressive disease (PD). There was a significant difference in OS between these SD and PD patients (*P* = 0.0312).

**Conclusions:**

Patients treated with sorafenib for HCC should be evaluated by mRECIST; RECIST 1.1 is preferable only for assessment of patients with lesions that are non-measurable according to mRESIST.

## Background

The Sorafenib Hepatocellular Carcinoma Assessment Randomized Protocol (SHARP) trial and phase III study conducted in the Asia–Pacific region proved survival benefits and good tolerability in the USA, Europe, and Asia–Pacific regions [1, 2]. Therefore, the current guidelines for the management of hepatocellular carcinoma (HCC) recommend sorafenib as first-line treatment for patients with advanced disease and well-preserved liver function (Child-Pugh A class) and state that there is no effective second-line treatment after sorafenib therapy; development of new regimens is required [3, 4].

Overall survival (OS) is the major endpoint used when investigating new treatment regimens for HCC and other cancers. Because earlier endpoints such as time to progression (TTP), progression-free survival (PFS), and response rate are used as guides for the early determination of whether to continue a particular therapy, these endpoints are appropriate in phase II trials, which aim at determining whether to launch larger scale clinical trials [5]. Response evaluation criteria in solid tumors (RECIST) is a well-established means of assessing tumor response and is used with confidence worldwide [6]; however, it was designed for assessing responses to cytotoxic agents and does not address measures of antitumor activity other than tumor shrinkage [7]. The use of RECIST to determine response rates to the treatment of HCC is controversial, especially when evaluating agents that reduce tumor vascularity and cell proliferation, resulting in stabilization of sizes of tumors despite central necrosis [5, 8, 9]. Thus, although sorafenib significantly improves OS compared with placebo, the response rate according to RECIST is only 2.0–3.3 % [1, 2].

Objective and precise criteria for evaluating treatment are required to determine whether to continue a particular treatment for HCC. A panel of experts on HCC convened by the European Association for the Study of the Liver (EASL) has therefore amended the response criteria to take tumor necrosis into account [10]. They recommended using contrast-enhanced radiologic imaging to identify viable lesions, which they defined as those that take up contrast agent in the arterial phase of dynamic studies, thus more accurately assessing treatment response [10]. As a result of their recommendations, a group from the American Association for the Study of Liver Diseases provided a common framework for clinical trials in patients with HCC by modified the RECIST guidelines (mRECIST), including incorporating the concept of viable tumor proposed by the EASL guidelines [8].

Some studies evaluating treatment responses to targeted agents have reported that mRECIST identifies more treatment responders and stratifies for OS better than RECIST in patients treated with sorafenib. However, these studies were not large (53–66 patients) [11–13]. Additionally, by drawing on data from a large database (>6500 patients), simulation studies, and reviews of published reports, RECIST has been further modified to produce the current revised RECIST version 1.1 (RECIST 1.1) [14].

The aim of this study was to identify the most appropriate assessment criteria for evaluating sorafenib treatment for HCC. To achieve this, we assessed the associations between responses as evaluated by conventional RECIST 1.1 and mRECIST and survival data to determine which criteria more accurately identified responders and predicted prognoses of responding patients.

## Methods

### Study design and selection of patients

This multicenter, retrospective, observational study aimed to assess tumor responses and prognoses of patients with HCC treated with sorafenib at four institutes of the Kanagawa Liver Study Group in Japan. The primary endpoint was to compare the ability of RECIST 1.1 and mRECIST to predict prognosis. All data, including patients’ histories, clinical characteristics, laboratory data, and radiological information were collected retrospectively from each institution.

The common eligibility criteria were as follows: (1) Eastern Cooperative Oncology Group (ECOG) performance status (PS) score of two or less; (2) Child–Pugh liver function class A or B; and (3) aged 20 years or older. The exclusion criteria were as follows: (1) previously treated with molecular-targeted therapies; (2) not treated with a single agent; (3) curative surgery performed after sorafenib treatment; and (4) responses to treatment not assessed by computed tomography (CT) scan.

This retrospective study was approved by the Ethical Committee of the Kitasato University (Kanagawa, Japan).

### Diagnosis

All patients had at least one index lesion measuring 1 cm or larger in diameter (i.e., a target lesion) at baseline. Confirmation of the diagnosis of HCC was obtained by observing the typical features of arterial enhancement followed by washout during the portal venous phase on dynamic scan or pathologically based on the American Association for the Study of Liver Diseases guidelines [4].

### Treatment regimens

Patients in this study were treated with sorafenib (Nexavar, Bayer Healthcare Pharmaceuticals) orally as a monotherapy. The standard dose was 400 mg (consisting of two 200 mg tablets) twice daily. Treatment interruptions and dose reductions (first 400 mg twice daily, then 400 mg once daily, and finally 400 mg every 2 days) were permitted for adverse events. In some patients who had poor liver function or were aged ≥75 years, the initial dose was only half of the standard dose (400 mg once daily). The following were assessed as treatment failure: (1) an unmanageable adverse event that required termination of therapy; (2) disease progression (according to either RECIST 1.1 or mRECIST); (3) liver function dwindled to Child-Pugh class C; (4) deterioration of ECOG PS score to four; and (5) withdrawal of consent to therapy.

### CT scan protocol

CT images were obtained after injection of 100–135 mL of iodine contrast (300 or 350 mg/mL) at an average flow rate of 3.0 mL/s using an automatic power injector. Slice collimation was 5 mm in the arterial and portal phases. Arterial phase imaging was performed 15–19 s after achievement of 200 HU attenuation of the descending aorta measured using a bolus tracking method. There was a 30- to 35-s delay between the arterial phase and portal venous phase acquisition.

### Evaluation of treatment response

Tumor measurements were performed on CT scans obtained within 1 month of commencing treatment and every 1–2 months during treatment.

Tumor responses were evaluated separately according to the best response according to RECIST 1.1 and mRECIST in a non-blinded fashion, by an oncologist and a radiologist who were both specialists in liver cancer. The target lesions were defined by both physicians on their pretreatment CT scans of all patients. Each physician then made separate measurements and determined the tumor responses. In cases of disagreement, a consensus was reached. Target lesions were defined as the whole lesion for RECIST assessment and as the contrast-enhanced portion of the lesion in the arterial phase for mRECIST assessment. For evaluation according to RECIST, complete response (CR) was defined as the absence of all target lesions; partial response (PR) and progressive disease (PD) as a greater than 30 % decrease and a greater than 20 % increase, respectively, in the sum of the longest diameters of the target lesions; and stable disease (SD) as neither PR nor PD [14]. However, for evaluation according to mRECIST, CR was defined as the absence of arterially enhanced areas in all target lesions; PR and PD as the same degree of decrease and increase as in the RECIST criteria, these sums being those of the diameters of arterial enhanced areas in all target lesions rather than the sums of the diameters of the whole target lesions size; and SD as neither PR nor PD. When evaluating according to mRECIST, patients with no enhanced lesions were classified as non-measurable. The greatest variation (maximum decrease or minimum increase) in the sum of the greatest lesion dimensions for each patient were also recorded [3, 8].

Adverse events were graded according to the National Cancer Institute’s Common Terminology Criteria for Adverse events, version 3.0 or 4.0.

### Identification of target and non-target lesions

At baseline, target lesions were defined as those largest diameters of 1 cm or more and non-target lesions as those with largest diameters of <1 cm. In accordance with RECIST 1.1 recommendations, a maximum of two lesions per organ and five lesions in total were selected for assessment of treatment response [6]. The same number of lesions were selected for assessment by mRECIST. Macroscopic vascular invasions were considered as non-target lesions. Extrahepatic lesions were considered as target lesions. Lesions with no arterial enhancement that were therefore not measurable by mRECIST were considered as non-target lesions when evaluating by mRECIST only [3, 8, 13].

### Statistical analysis

Continuous variables are presented as medians and ranges and categorical variables as percentages. Between-group comparisons of variables were performed using the Mann–Whitney U test for continuous variables and the Chi square or Fisher’s exact test for categorical variables. Survival times were measured from the start of sorafenib initiation. OS ended at the time of death or was censored at the time of the last follow-up visit. Duration of sorafenib treatment ended at the time of the last administration of sorafenib for any reason or was censored at the time of the last follow-up visit. Survival analyses were performed using the Kaplan–Meier method and compared using the log-rank test. A Cox proportional hazards model was used to determine independent prognostic factors for OS after adjustment for confounding factors. All reported *P* values are two-sided, *P* values <0.05 being considered statistically significant. Dr SPSS Base 11.0j (SPSS Inc., Chicago, IL, USA) for Windows was used to perform all computations, and two-tailed *P* values <0.05 were considered to indicate statistical significance.

## Results

### Patients and baseline characteristics

**Fig. 1:**
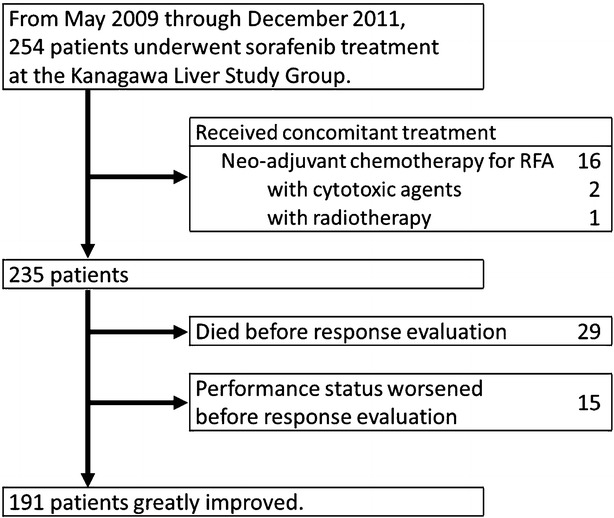
Flow diagram of study enrollment

Figure 1 shows a flow diagram of patient recruitment for this study. Between May 2009 and December 2011, 254 patients underwent sorafenib treatment at four institutes of the Kanagawa Liver Study Group. Of these 254 patients, 63 were excluded, 19 (9.9 %) because they had received concomitant treatment (16 with radio-frequency ablation, two with cytotoxic agents, and one with radiotherapy) and 44 (23 %) because post-treatment CT scans had not been performed (because of death in 29 patients and worsening of PS in 15). Thus, 191 patients were finally included in the current study. Treatments prior to sorafenib were as follows; surgical resection in 11 patients, percutaneous locoregional treatments in 20, arterial chemoembolization in 117, arterial chemotherapy in 16, systematic chemotherapy in two, and internal radiation in seven. Eighteen patients had not undergone any treatment before sorafenib.

Baseline characteristics are shown in Table 1. The standard dosage of sorafenib was initially administered to 126 patients (66 %) and the half-dose regimen to the remaining 65 (34 %).

**Table 1:** Baseline characteristics of study subjects

Characteristics	Study cohort (*n* = 191)
Age (years): median (range)	72 (34–88)
Male gender: no. (%)	149 (78)
Cause of liver disease: no. (%)	
HC	112 (59)
HB	35 (18)
HC + HB	4 (2)
Others	40 (21)
Child–Pugh class: no. (%)	
A/B	179/12 (94/6)
ECOG PS: no. (%)	
0	141 (74)
1	47 (25)
2	3 (1)
BCLC stage: no. (%)	
A	11 (6)
B	85 (44)
C	95 (50)
Prior treatment: no. (%)	
Resection	13 (7)
Radio frequency ablation	22 (11)
Transarterial chemoembolization	137 (72)
None	19 (10)
Macroscopic vascular invasion: no. (%)	51 (27)
Extrahepatic spread: no. (%)	56 (29)
Reduced initiation dose: no. (%)	65 (34)
Laboratory data: median (range)	
Hemoglobin (g/dL)	12.5 (7.7–18.0)
Platelet count (10^4^/μ)	12.3 (3.5–47.5)
Aspartate aminotransferase (IU/L)	55 (13–354)
Alanine aminotransferase (IU/L)	44 (10–238)
Albumin (g/dL)	3.7 (2.4–4.7)
Total bilirubin (mg/dL)	0.8 (0.3–2.6)
AFP (ng/mL)	81 (1.6–409,000)
DCP (mAU/mL)	332 (8–893,000)

*AFP* alpha-fetoprotein, *BCLC* Barcelona clinic liver cancer stage, *DCP* des-gamma carboxy prothrombin, *ECOG* Eastern Cooperative Oncology Group, *HB* hepatitis B virus, *HC* hepatitis C virus, *NBNC* non-B and non-C hepatitis virus, *PS* performance status

### Safety and tolerability

The median duration of follow-up in this cohort was 9.7 months [range 1.0–31.9 months; 95 % confidence interval (95 % CI) 8.2–11.2]. The median duration of sorafenib treatment was 2.0 months (range 0.7–12.5 months; 95 % CI 1.9–2.1). Seventy-three patients (38 %) stopped sorafenib treatment because of adverse events, 83 (43 %) because of disease progression and one patient was censored for changing anticancer agent before disease progression. Table 2 shows drug-related adverse events.

**Table 2:** Drug-induced adverse events according to the common terminology criteria for adverse events version 3.0 or 4.0

	All grades	Grade 3 or 4
	No. (%)	No. (%)
Adverse events	181 (94.8)	103 (53.9)
Hand-foot skin reaction	120 (62.8)	38 (19.9)
Elevated AST	114 (59.7)	26 (13.6)
Elevated ALT	103 (53.9)	14 (7.3)
Fatigue	98 (51.3)	13 (6.8)
Appetite loss	84 (44.0)	20 (10.5)
Diarrhea	76 (39.8)	12 (6.3)
Hypertension	73 (38.2)	11 (5.8)
Skin rash	40 (20.9)	5 (2.6)
Voice changes	23 (12.0)	2 (1.0)
Alopecia	23 (12.0)	0 (0.0)
Gastrointestinal bleeding	4 (2.1)	1 (0.5)
Variceal bleeding	3 (1.6)	2 (1.0)

*ALT* alanine aminotransferase, *AST* aspartate aminotransferase

### Treatment responses

Table 3 shows the best responses as assessed by both RECIST 1.1 and mRECIST. According to RECIST 1.1, the response rate (defined as the percentage of CR or PR patients) was 7.8 % (15/191) and the disease control rate (defined as percentage of CR + PR + SD patients) was 53.4 % (102/191). However, according to mRECIST, the response rate was 14.3 % (25/191) and the disease control rate 55.4 % (106/191). Evaluation of response to treatment by RECIST1.1 and mRECIST corresponded in 162 patients (84.8 %) (CR/PR/SD/PD: 4/11/70/77, respectively). Ten patients evaluated as non-responders by RECIST1.1 were evaluated by mRECIST as responders (one patient’s response changed from SD to CR, eight from SD to PR, and one from PD to PR). Sixteen patients were not evaluated by mRECIST because they had no measurable target lesions (no enhanced lesions). Eight of these patients were evaluated by RECIST1.1 as having SD and eight as PD.

**Table 3:** Treatment evaluation according to response evaluation criteria in solid tumors (RECIST) version 1.1 and modified (m) RECIST

	RECIST 1.1				Total (mRECIST)
	CR	PR	SD	PD	
mRECIST					
CR	4	0	1	0	5
PR	0	11	8	1	20
SD	0	0	70	2	72
PD	0	0	1	77	78
Non-measurable	0	0	8	8	16
Total (RECIST 1.1)	4	11	88	88	191

*CR* complete response, *PD* progressive disease, *PR* partial response, *SD* stable disease

### Survival analysis

**Fig. 2:**
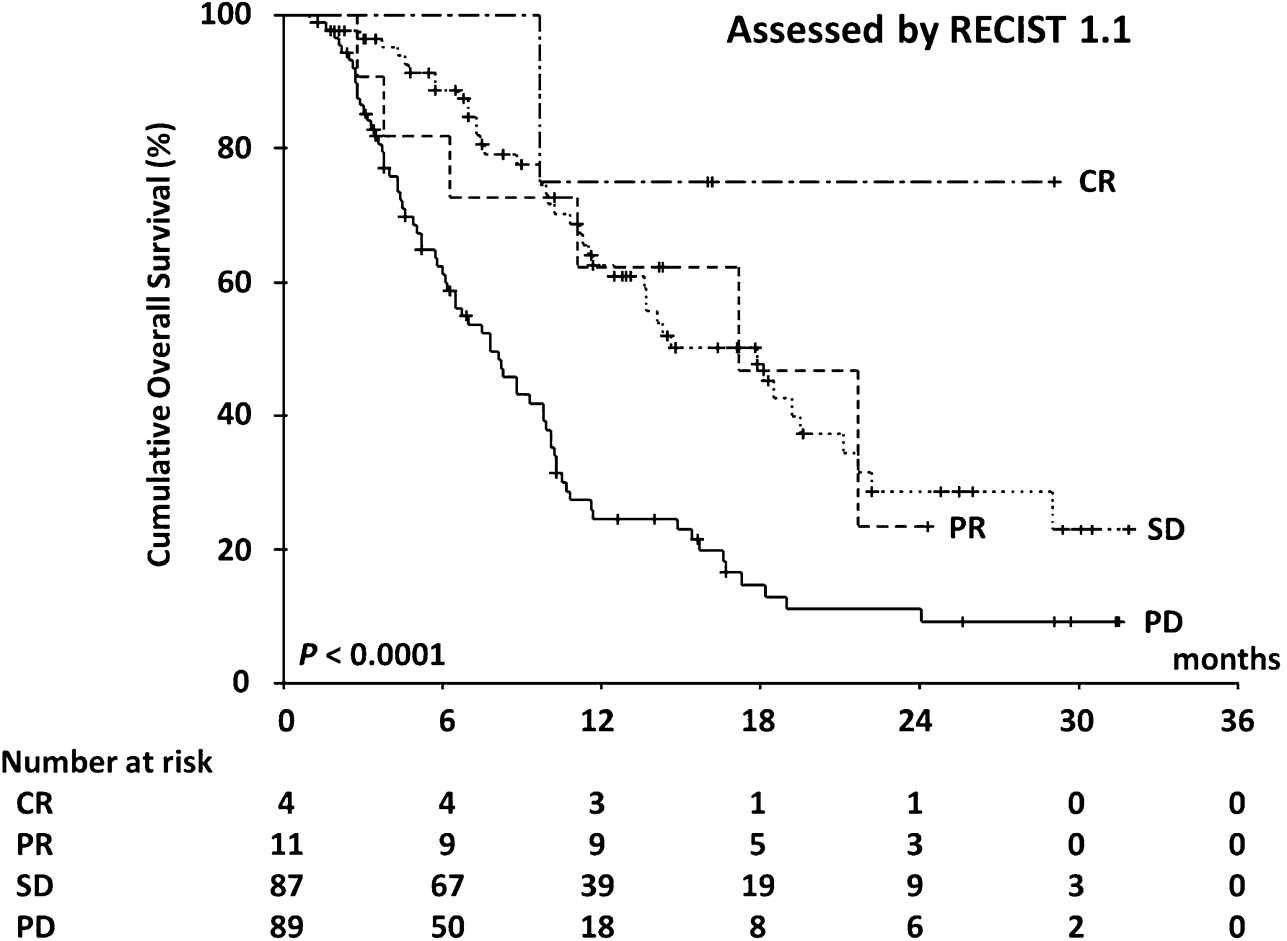
Kaplan–Meier analysis of overall survival. The log-rank test showed significant differences between the four groups (*P* < 0.0001) categorized according to RECIST 1.1

**Fig. 3:**
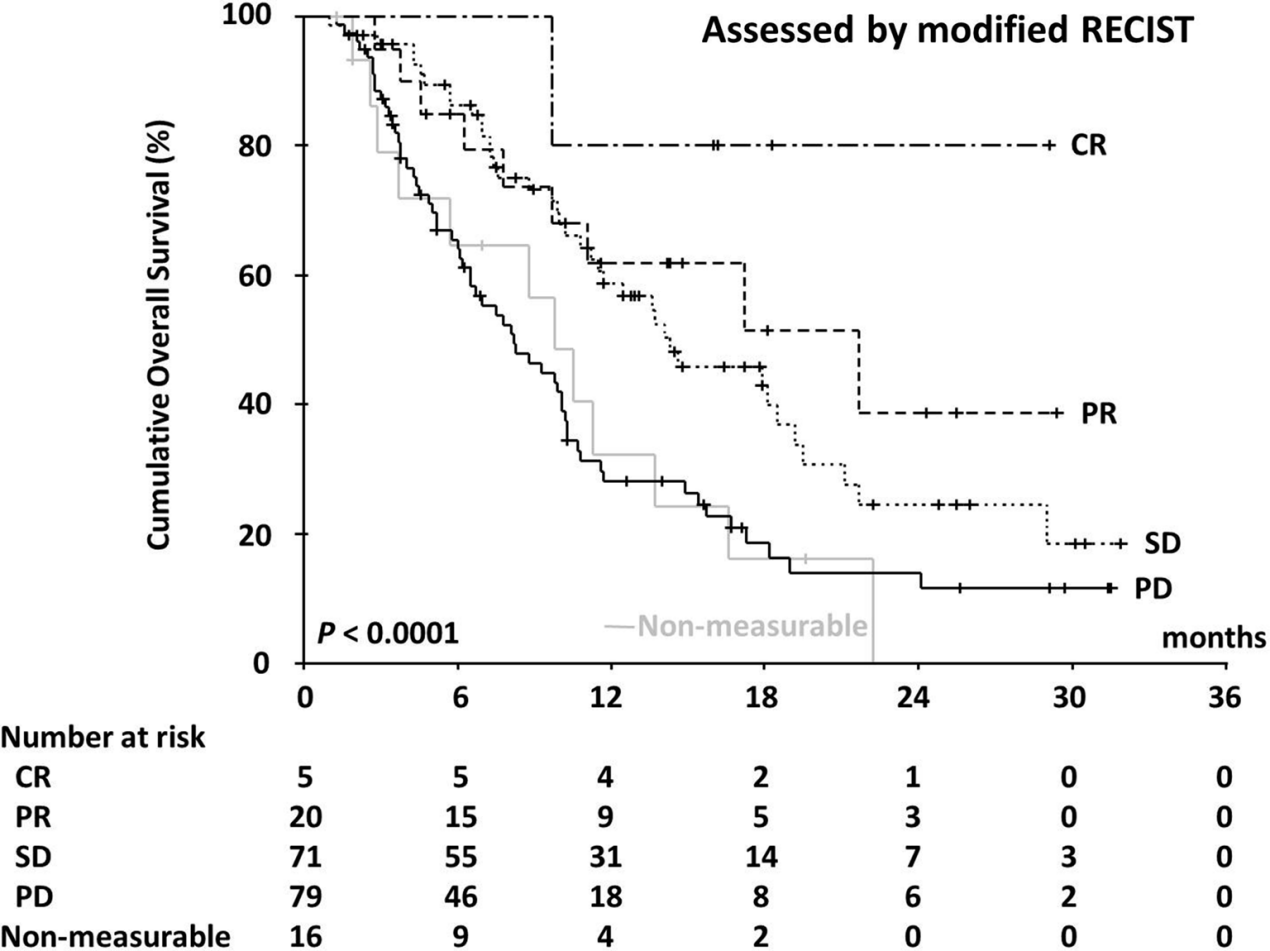
Kaplan–Meier analysis of overall survival. The log-rank test showed significant differences between the four groups (*P* < 0.0001) categorized according to mRECIST

**Fig. 4:**
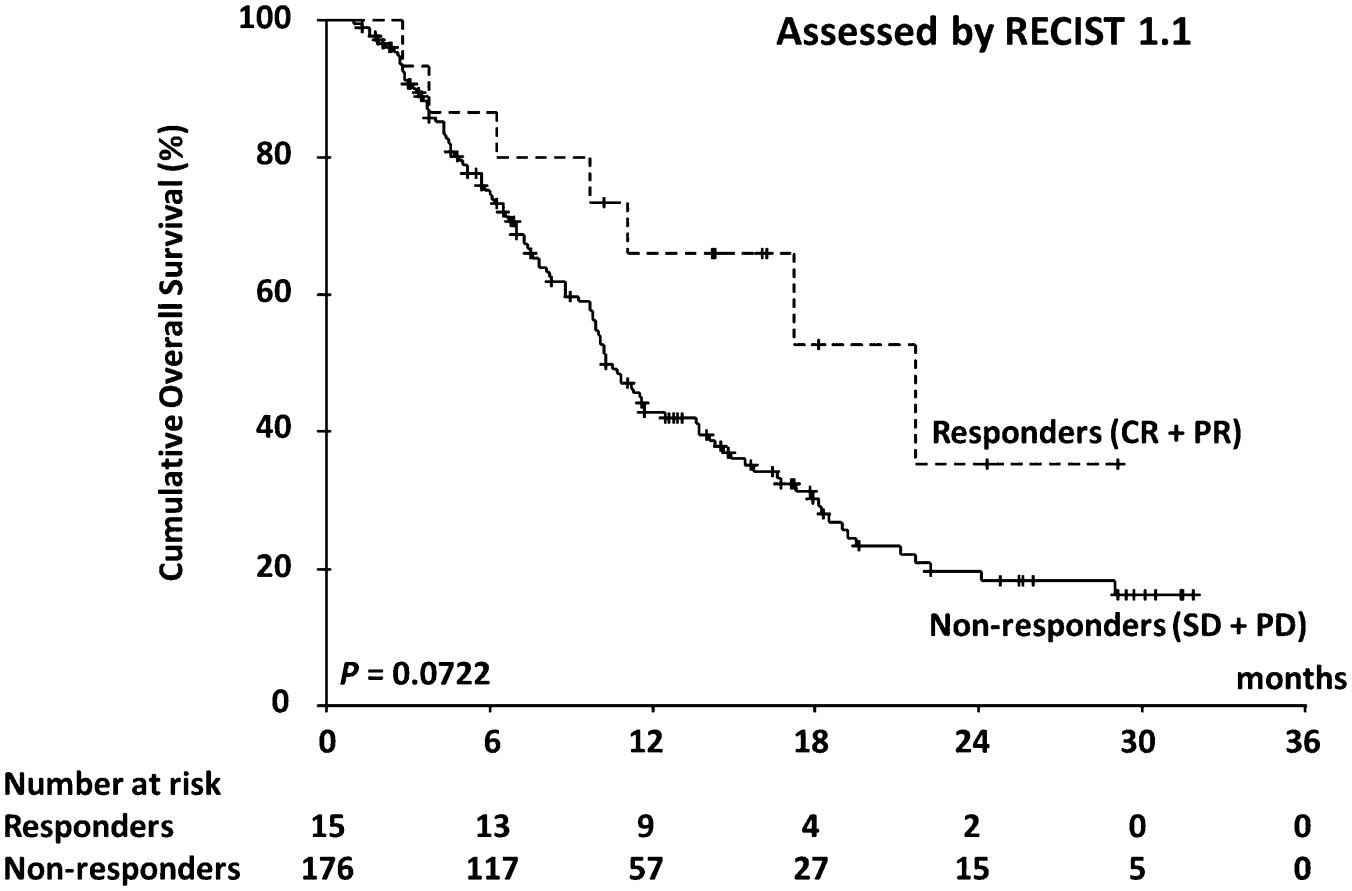
Kaplan–Meier analysis of overall survival. The log-rank test did not show significant differences between responders and non-responders classified according to RECIST 1.1 (*P* = 0.0722)

**Fig. 5:**
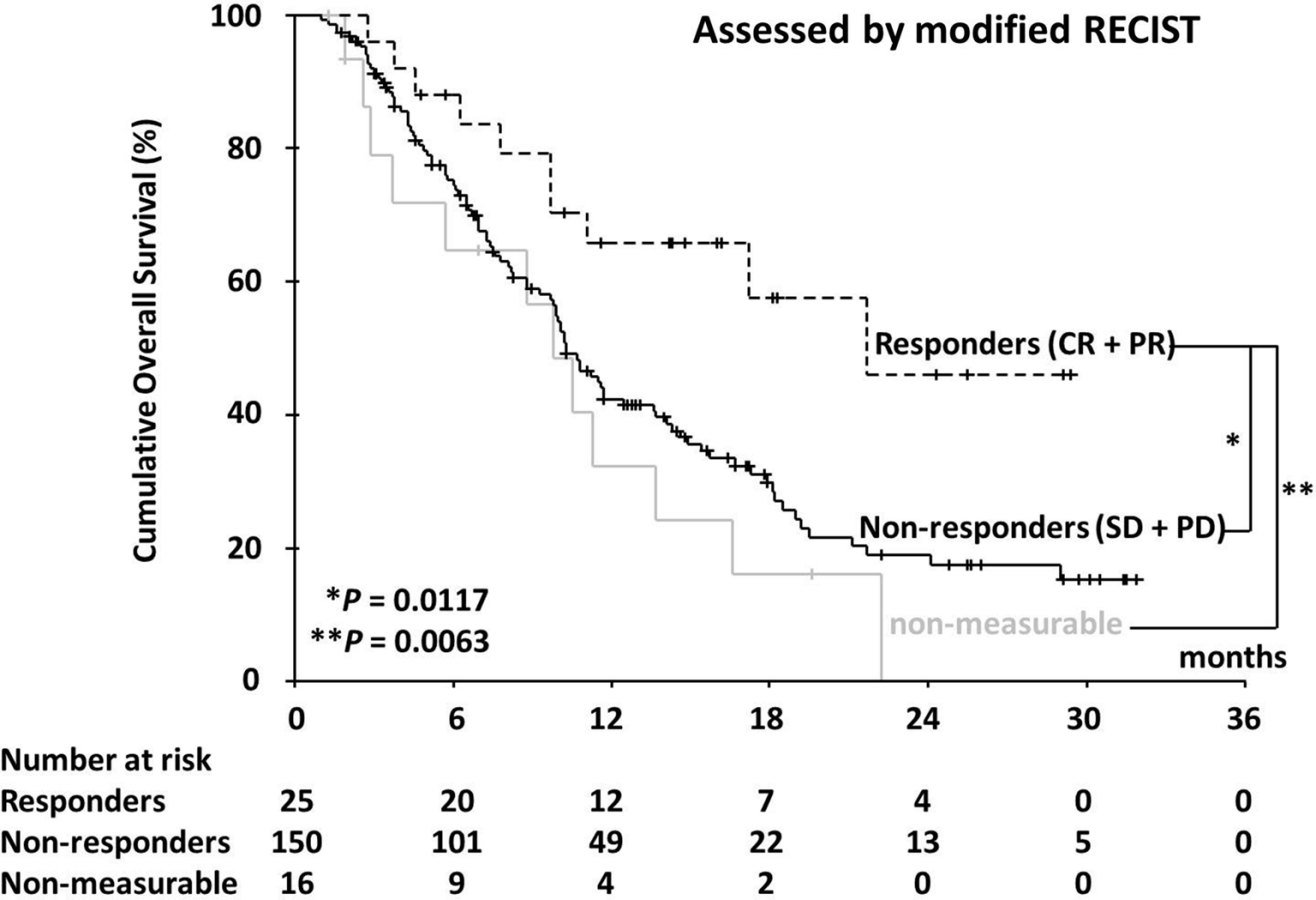
Kaplan–Meier analysis of overall survival. The log-rank test showed significant differences between responders and non-responders classified according to mRECIST (*P* = 0.0117)

**Fig. 6:**
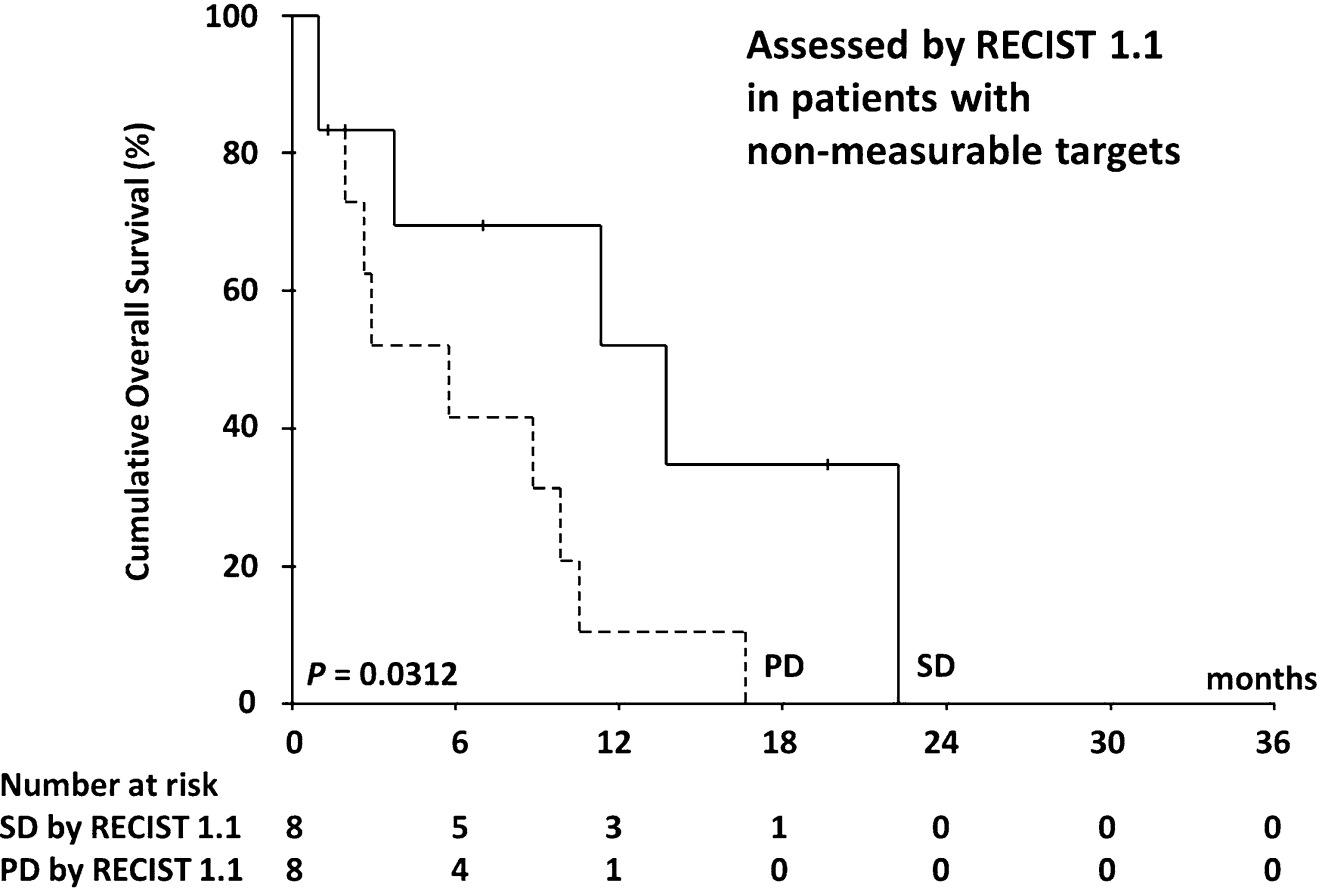
Kaplan–Meier analysis of overall survival in small cohort of patients with non-measurable lesions by mRECIST (n = 16). The log-rank test showed significant differences between progressive disease (PD) (n = 8) and stable disease (SD) groups (n = 8) as determined by RECIST 1.1 (P = 0.0312)

In all, 120 patients (62.8 %) died during the study period. The median OS was 10.8 months (range 1.0–31.9 months; 95 % CI 9.6–12.0) and the 1-year survival rate was 44.8 %. Figures 2 and 3 show OS curves for the four response categories as evaluated by RECIST1.1 and mRECIST, whereas Figs. 4 and 5 show a comparison of OS for responders and non-responders as evaluated by RECIST 1.1 and mRECIST. Patients classified as responders by mRECIST had significantly better OS than non-responders (Fig. 5; *P* = 0.0117). However, the OS of those classified as responders by RECIST1.1 did not differ significantly from that of non-responders (Fig. 4). Although the treatment responses of 16 patients (8.4 %) could not be assessed by mRECIST because they had no enhanced lesions (Table 3), eight of them were assessed as SD and eight as PD by RECIST1.1. These subgroups had significantly different OS (Fig. 6; median OS = 13.7 months versus 5.7 months, respectively; *P* = 0.0312).

## Discussion

In this study, we compared assessment of response to sorafenib by RECIST 1.1 and mRECIST in patients with advanced HCC to determine which criteria more accurately predict prognosis. Although we found that both RECIST1.1 and mRECIST could distinguish PD group from controlled disease, mRECIST identified approximately double the number of responders that RECIST 1.1 did; consequently, the response rate according to mRECIST exceeded 10 % whereas that determined by RECIST 1.1 did not. Responders according to mRECIST had a significantly better OS than nonresponders; this was not true for RECIST. The potential benefits of the more accurate identification of responders by mRECIST include the following: (1) a partial response to therapy can serve as a surrogate marker of clinical benefit [7, 15]; and (2) response rates exceeding 10 % in phase II studies of single-agent non-cytotoxic drug therapy are generally an indicator of survival advantages in phase III studies [7]. Therefore, we believe that mRECIST is superior to RECIST 1.1 for assessing antitumor effects in phase II studies of targeted therapies for HCC.

This study showed a good overall response rate to sorafenib (CR + PR; 7.8 %) compared with published prospective trials (2.0–3.3 %) [1, 2]. However, in one study, 9.6 % of Japanese patients with HCC treated with sorafenib were reportedly classified by RECIST1.1 as responders [16]; these authors suggested there may be a racial difference concerning gene mutations that influence the response rate, similar to the epidermal growth factor receptor mutation for gefitinib [17].

A number of previous clinical studies of HCC treatment have demonstrated that RECIST criteria, which do not reflect tumor vascularization, do not accurately mirror the extent of tumor necrosis induced by chemoembolization, transcatheter chemotherapies, and percutaneous locoregional therapies [18–20]. These treatments can be demonstrated to have conferred benefit if tumor vascularization is taken into account by identifying viable tumor by its enhancement with contrast agent in the arterial phase of a dynamic CT [8]. Llovet et al. claimed that assessment of reduction in volume of viable tumor using contrast-enhanced radiological imaging is the optimal means of assessing treatment response [5]. Thus, MRECIST is more accurate than RECIST for evaluating responses of HCC, which are commonly hypervascular.

According to mRECIST, non-enhanced tumor is not measurable. Assessment of such lesions is important because evaluation of response may otherwise be incomplete. Among several additional radiologic criteria aimed at evaluating tumor viability after treatment with targeted agents, Choi et al. proposed basing such criteria on target density as determined by measuring the CT attenuation coefficient; this approach could be useful for evaluating HCC treatment [13, 21–25]. However, our subgroup analysis indicated that assessment by conventional RECIST adequately predicts prognosis in patients with non-enhancing atypical lesions without using Choi’s criteria because, as Llovet at el. reported, the conventional RECIST criteria are applicable to non-enhancing atypical lesions [5]. However, measurement of the size of the tumor alone has limitations because it cannot distinguish tumor proliferation from necrosis, as previously described [26]. Additionally, magnetic resonance imaging and measurement of serum alfa-fetoprotein levels are useful for clinical evaluation of non-enhancing atypical lesions and may differentiate tumor proliferation from necrosis in such tumors [12, 21, 27–30].

### Limitations

In this multicenter retrospective study, post-sorafenib treatment and reduction in dosage of sorafenib were not standardized. In addition, assessment of response to treatment may have varied according to the CT scan procedures and interpretations in each institute. However, we believe that our findings do reflect clinical outcomes of both sets of criteria because this multicenter study was able to enroll many more patients than previously reported studies [11–13].

## Conclusions

Patients treated with sorafenib for HCC should be evaluated by mRECIST; RECIST 1.1 is preferable only for assessment of patients with lesions that are non-measurable according to mRECIST.

## Abbreviations

CI: confidence interval
CR: complete response
CT: computed tomography
EASL: European Association for the Study of the Liver
ECOG: Eastern Cooperative Oncology Group
HCC: hepatocellular carcinoma
mRECIST: modified response evaluation criteria in solid tumors
OS: overall survival
PFS: progression-free survival
PD: progressive disease
PR: partial response
PS: performance status
RECIST: response evaluation criteria in solid tumors
SD: stable disease
SHARP: The Sorafenib Hepatocellular Carcinoma Assessment Randomized Protocol
TTF: time to treatment failure
TTP: time to progression
TTS: time to treatment stop
